# Supplementary material for: Spin-defect qubits in two-dimensional transition metal dichalcogenides operating at telecom wavelengths
Source: Nat Commun. 2022 Dec 6;13:7501. doi: 10.1038/s41467-022-35048-0 (PMC9723673; doi:10.1038/s41467-022-35048-0)
Supplement: Supplementary file 1 — Supplementary information [file 41467_2022_35048_MOESM1_ESM.pdf]

Supplementary information

Spin-defect qubits in two-dimensional transition metal dichalcogenides operating at telecom wavelengths

Yeonghun Lee[1,2,†], Yaoqiao Hu[1], Xiuyao Lang[1], Dongwook Kim[1], Kejun Li[3], Yuan Ping[4], Kai-Mei C. Fu[5,6], and Kyeongjae Cho[1,*]

[1] Department of Materials Science and Engineering, The University of Texas at Dallas, Richardson, TX 75080, USA

[2] Department of Electronics Engineering, Incheon National University, Incheon 22012, Republic of Korea

[3] Department of Physics, University of California, Santa Cruz, CA 95064, USA

[4] Department of Chemistry and Biochemistry, University of California, Santa Cruz, CA 95064, USA

[5] Department of Physics, University of Washington, Seattle, WA 98195, USA

[6] Department of Electrical and Computer Engineering, University of Washington, Seattle, WA 98195, USA

Corresponding authors

† Email: y.lee@inu.ac.kr

* Email: kjcho@utdallas.edu

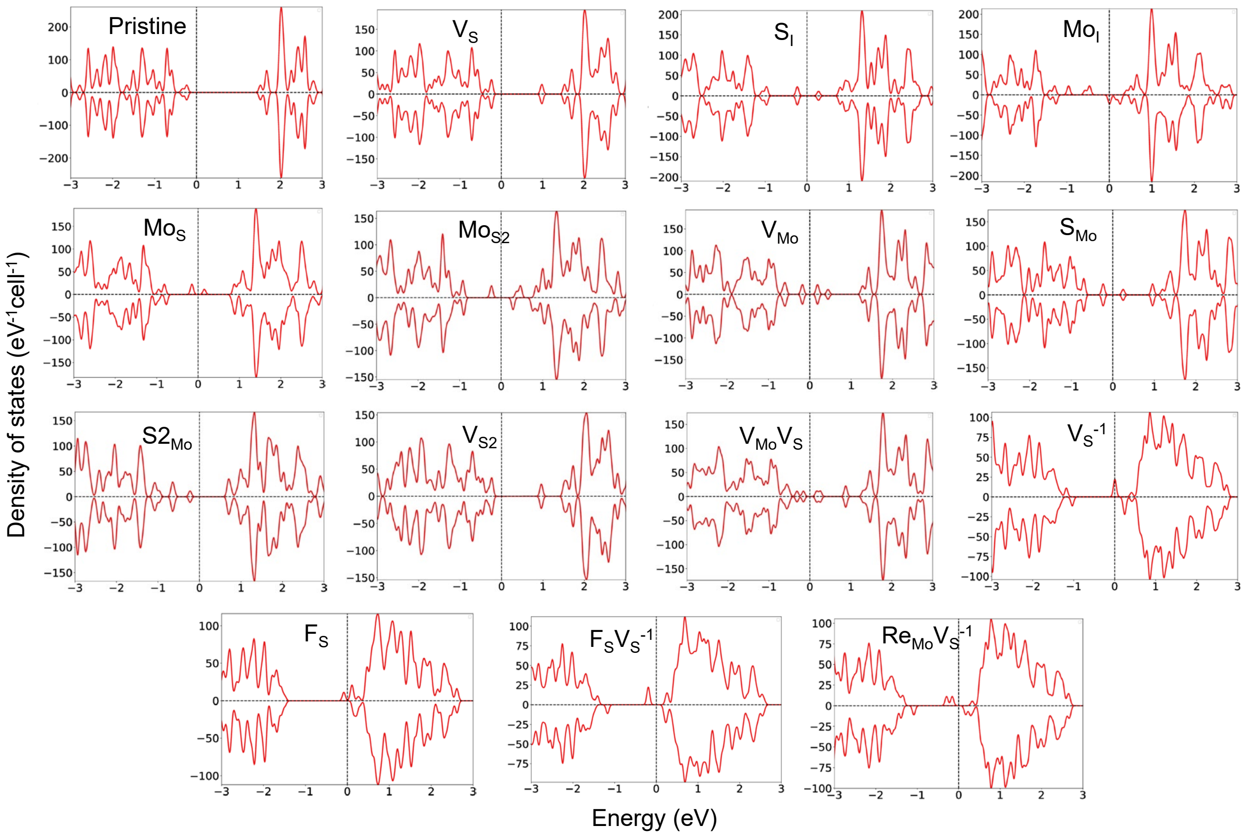

Supplementary Figure 1: Identification of the spin-triplet ground-state. Densities of states as a function of energy relative to the Fermi level for intrinsic (or native) and extrinsic defects in MoS_2 are calculated without using the hybrid functional for quick screening. Donor-vacancy defect complexes ($F_SV_S^{-1}$ and $Re_{Mo}V_S^{-1}$) exhibit the spin-triplet ground state but do not have spin-conserving intradefect optical transition without ionization of the defects. Mo_S turns out to be the spin-triplet ground state with well-separated defect energy levels.

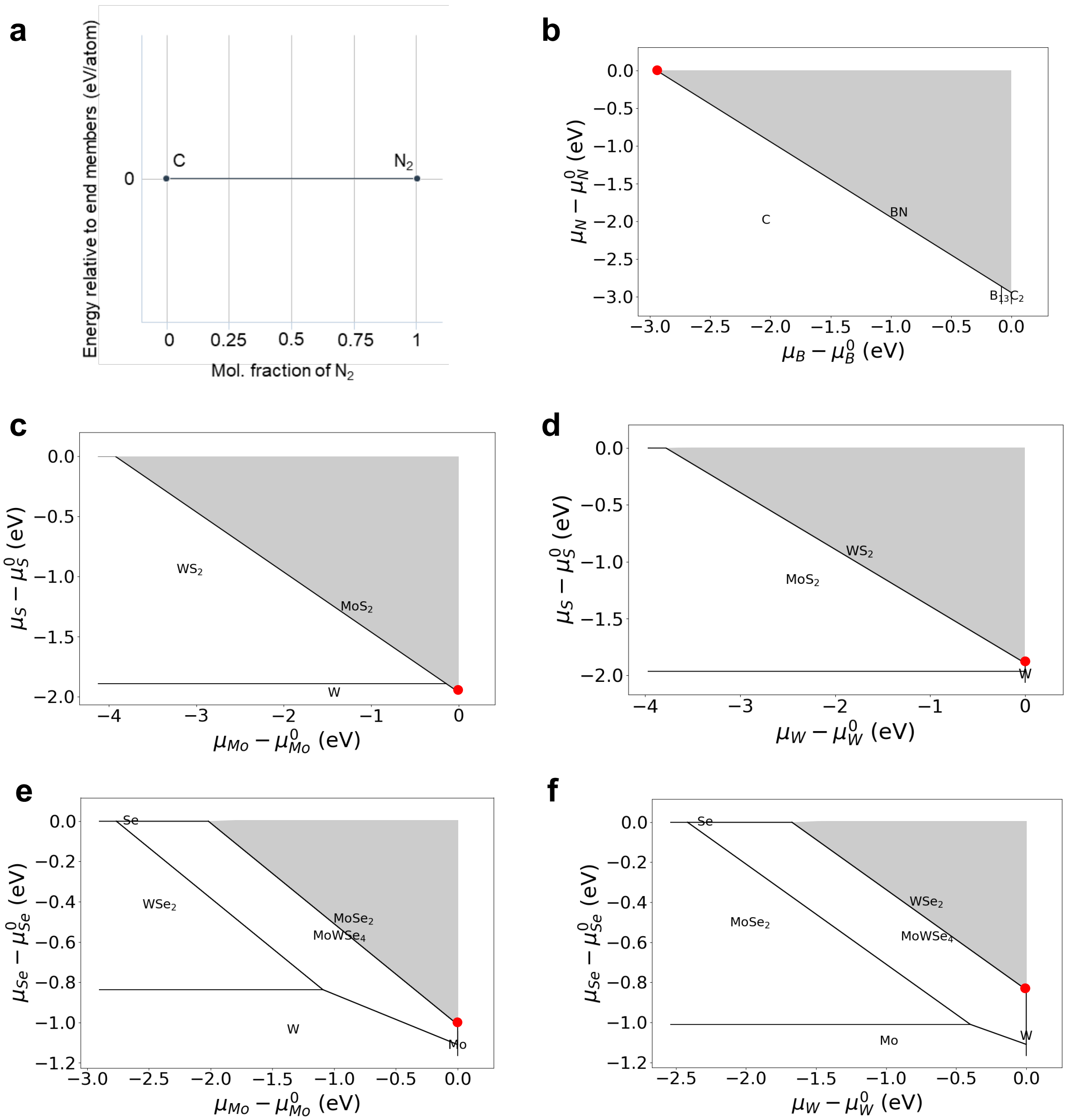

Supplementary Figure 2: Chemical potential range of chemical species. Phase diagrams provided by Materials Project[1] to determine chemical potentials for the formation energy diagrams. (a) Phase diagram of a binary compound C-N. Phase stability diagrams of ternary compounds (b) B-N-C in the B-N chemical potential space, (c) Mo-S-W in the Mo-S chemical potential space, (d) W-S-Mo in the W-S chemical potential space, (e) Mo-Se-W in the Mo-Se chemical potential space, and (f) W-Se-Mo in the W-Se chemical potential space. Red dots indicate the N-rich condition and the host metal rich conditions, showing lower defect formation energy.

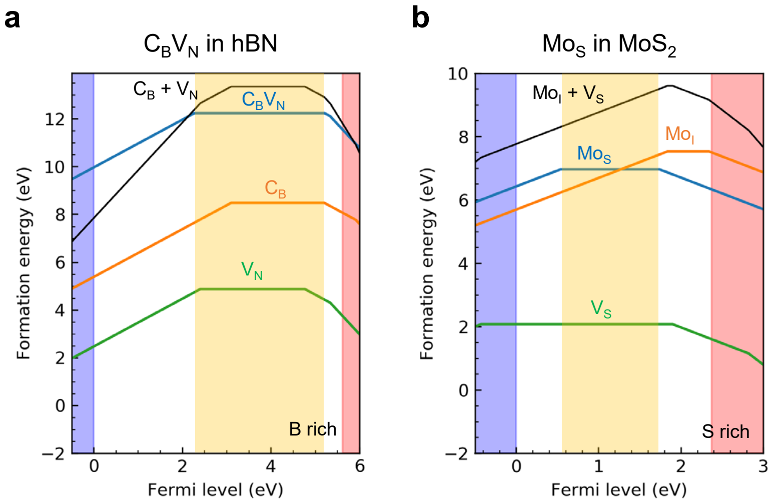

Supplementary Figure 3: Defect formation energy diagrams in other conditions. Defect formation energy diagrams for (a) C_BV_N in monolayer hBN and (b) Mo_S in monolayer MoS_2 under different conditions with Figure 2(b,c). The B-rich shows higher formation energy of C_BV_N in hBN than the N-rich condition shown in Figure 2 (b). The S-rich condition shows higher formation energy of Mo_S. in MoS_2 than the Mo-rich condition shown in Figure 2 (c). This tendency will remain the same in the M_X defect family.

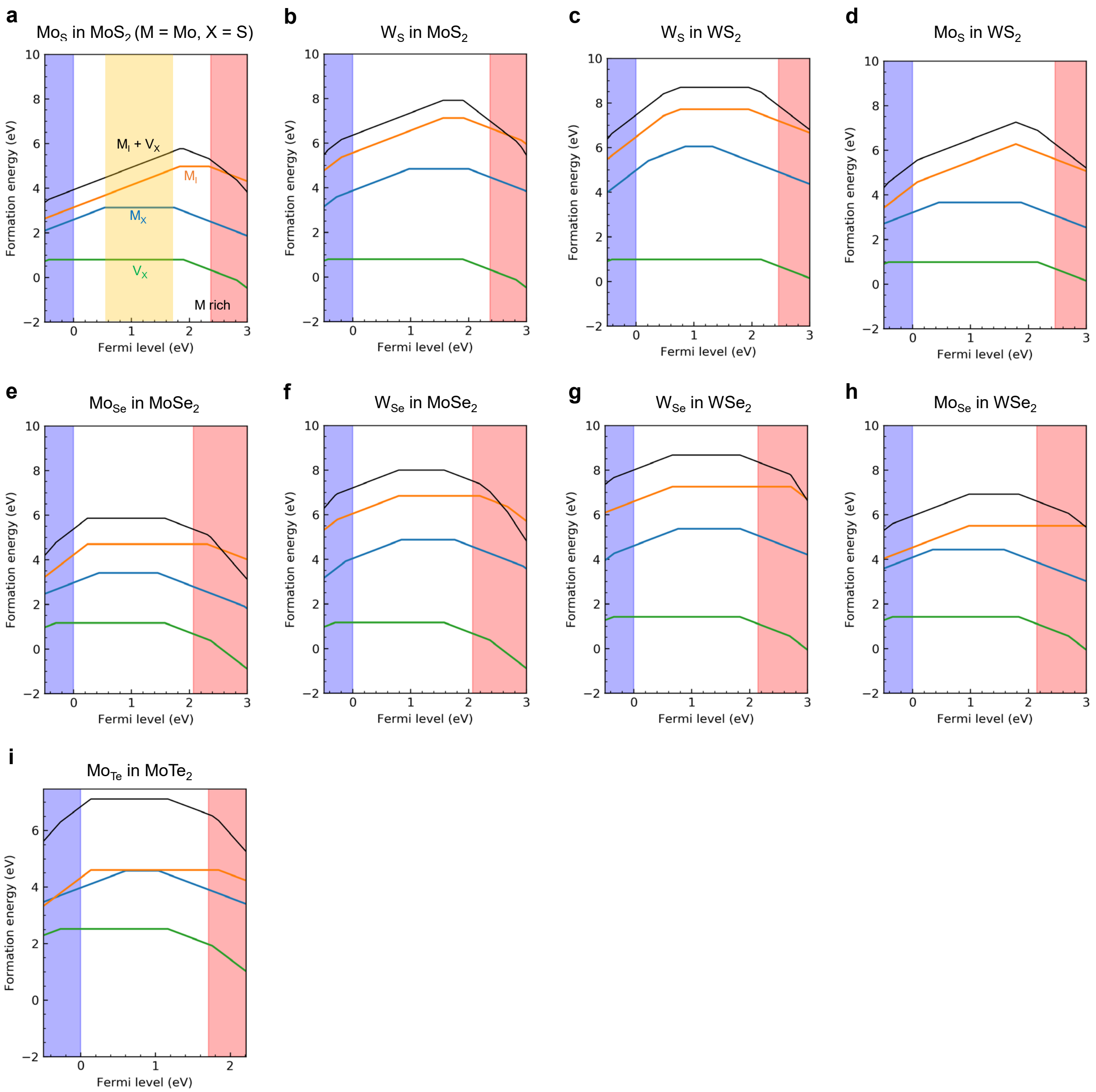

Supplementary Figure 4: Defect formation energy diagrams for all family. Defect formation energy diagrams for M_X, V_X, M_I, and two independent defects ($M_I + V_X$) in monolayer MX_2 under the host's M-rich condition. The formation energy of M_X defects is lower than the total formation energies of $M_I + V_X$, indicating that the formation of M_X defects is favorable when the system accommodates M_I and V_X defects.

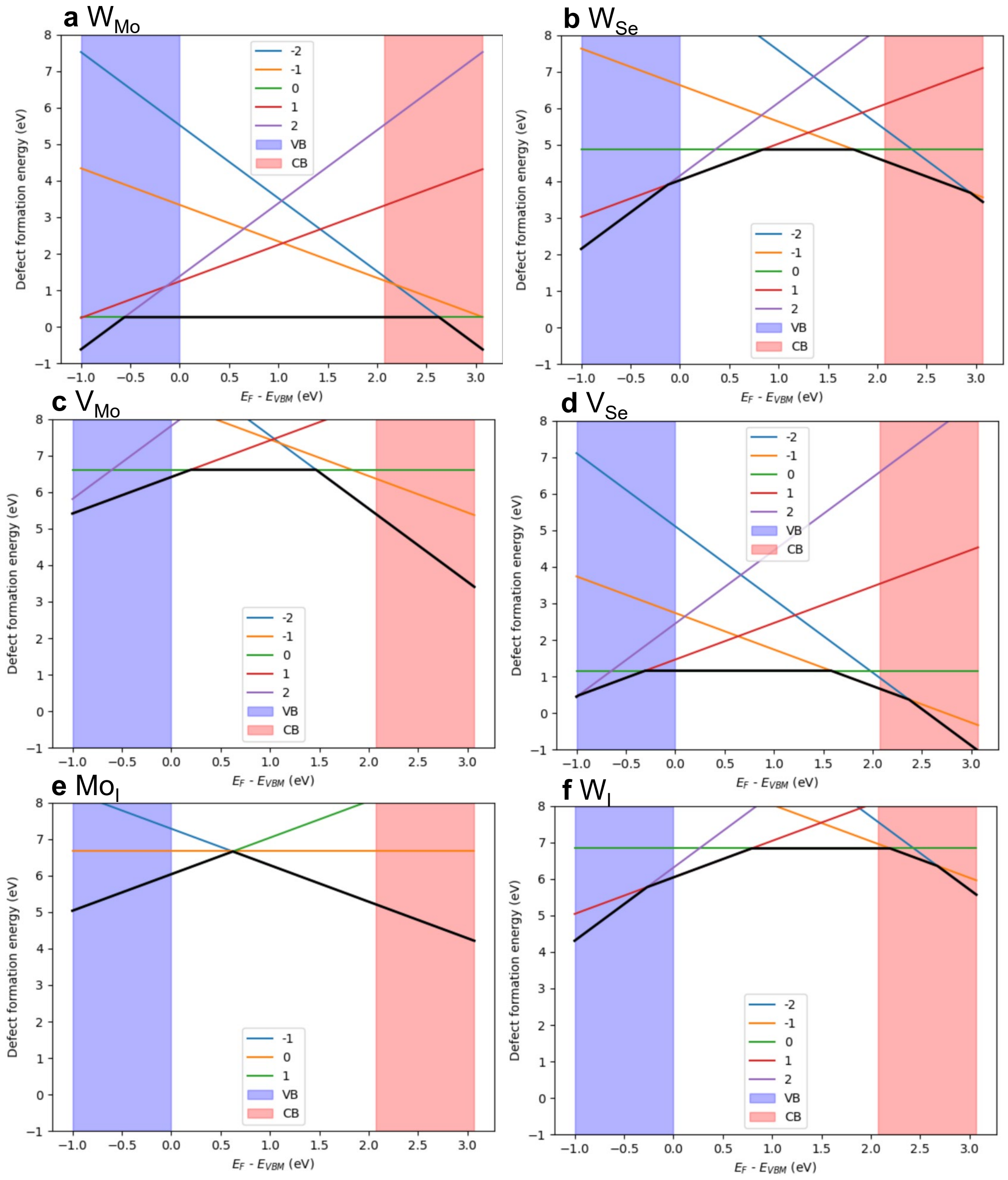

Supplementary Figure 5: Formation of competing defects. Defect formation energies of other competing defects with W_{Se} in $MoSe_2$ under the host's Mo-rich condition. V_{Se} is much easier to be formed than V_{Mo}, and the stability of W_I is almost the same as that of Mo_I. Thus, once we introduce W_I in the presence of abundant V_{Mo}, the W_{Se} complex can be readily formed.

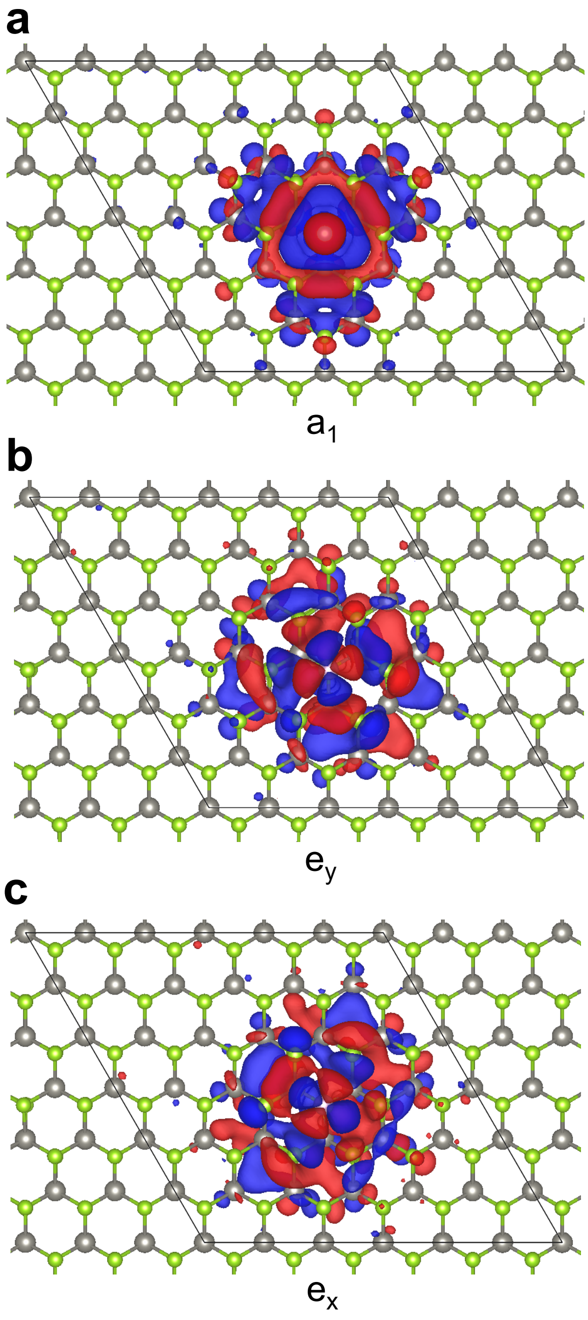

Supplementary Figure 6: Wavefunctions of the W_{Se} defect in $MoSe_2$. Real parts of wavefunctions of (a) a_1, (b) e_y, and (c) e_x states associated with W_{Se} in $MoSe_2$. The partial density of states analysis indicates that the e_x, e_y, and a_1 states are composed of $0.36d_{\mathrm{xy}} + 0.12d_{x^2-y^2}$, $0.12d_{\mathrm{xy}} + 0.36d_{x^2-y^2}$, $0.48d_{z^2} + 0.03S$, respectively.

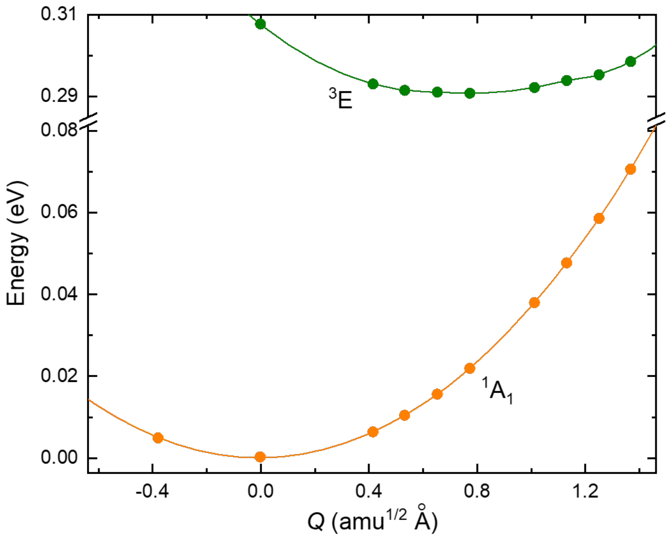

Supplementary Figure 7: Configuration coordinate diagrams for calculating the ISC transition rate. Configuration coordinate diagrams for ^{3}E and $^{1}A_1$ state of the W_{Se} defect in $MoSe_2$.

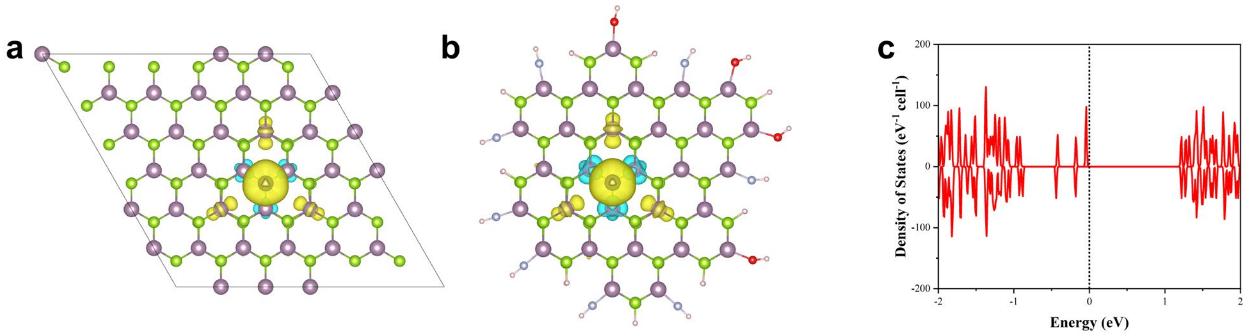

Supplementary Figure 8: Validation of the cluster model. Structures and spin densities for (a) periodic and (b) cluster W_{Se} defect in $MoSe_2$ (isosurface level = 0.00296 e/bohr^3). (c) Densities of states as a function of energy relative to the Fermi level for W_{Se} cluster using B3LYP functional.

Supplementary Table 1: Physical quantities extracted from configuration coordinate diagrams.

		Zero-field splitting tensors (GHz)			
Hosts	Defects	D_{xx}	D_{yy}	D_{zz}	D
Diamond	$N_C V_C^{-1}$	-0.95	-0.95	1.90	2.86
hBN	$C_B V_N$	-0.91	-6.29	7.18	10.77
MoS_2	Mo_S	-6.83	-6.83	13.67	20.51
	W_S	-4.48	-4.49	8.96	13.44
WS_2	W_S	-4.81	-4.81	9.63	14.44
	Mo_S	-7.21	-7.21	14.43	21.65
$MoSe_2$	Mo_{Se}	-6.37	-6.37	12.75	19.13
	W_{Se}	-4.14	-4.14	8.29	12.43
WSe_2	W_{se}	-4.29	-4.29	8.58	12.88
	Mo_{Se}	-6.60	-6.60	13.21	19.82
$MoTe_2$	Mo_{Te}	-0.16	-0.16	0.31	0.47

Supplementary Table 2: Calculated hyperfine tensors for $N_CV_C^{-1}$ in diamond, C_BV_N in hBN, and M_X in TMDs.

Hosts	Defects	Nuclear spin	Numbers of equivalent sites	Hyperfine tensors (MHz) (convention: $\lvert A_{zz}\rvert > \lvert A_{xx}\rvert > \lvert A_{yy}\rvert$)		
				A_{xx}	A_{yy}	A_{zz}
Diamond	$N_CV_C^{-1}$	^{14}N (I = 1, 99.632%)	1	-2.9	-2.6	-2.9
		^{15}N (I = 1/2, 0.368%)	1	4.1	3.6	4.1
		^{13}C (I = 1/2, 1.07%)	3	145.0	144.8	227.2
		^{13}C (I = 1/2, 1.07%)	6	14.2	14.1	19.9
hBN	C_BV_N	^{13}C (I = 1/2, 1.07%)	1	474.7	400.9	478.8
		^{10}B (I = 3, 19.9%)	1	24.9	22.2	26.4
		^{11}B (I = 3/2, 80.1%)	2	74.4	66.3	78.9
		^{14}N (I = 1, 99.632%)	2	7.3	7.2	9.9
		^{15}N (I = 1/2, 0.368%)	2	-10.3	-10.1	-13.9
MoS_2	Mo_S	^{95}Mo (I = 5/2, 15.92%)	1	-114.9	-37.5	-114.9
		^{97}Mo (I = 5/2, 9.55%)	1	-117.3	-38.3	-117.3
		^{95}Mo (I = 5/2, 15.92%)	3	16.3	9.4	18.8
		^{97}Mo (I = 5/2, 9.55%)	3	16.7	9.6	19.2
		^{33}S (I = 3/2, 0.76%)	6	13.1	12.9	15.4
	W_S	^{183}W (I = 1/2, 14.31%)	1	296.3	208.9	296.3
		^{95}Mo (I = 5/2, 15.92%)	3	12.5	6.5	12.6
		^{97}Mo (I= 5/2, 9.55%)	3	12.7	6.7	12.8
		^{33}S (I = 3/2, 0.76%)	6	15.9	14.7	17.2
WS_2	W_S	^{183}W (I = 1/2, 14.31%)	1	276.0	184.2	276.0
		^{183}W (I = 1/2, 14.31%)	3	-24.8	-20.3	-26.4
		^{33}S (I = 3/2, 0.76%)	6	16.1	15.5	17.3
	Mo_S	^{95}Mo (I = 5/2, 15.92%)	1	-102.7	-19.8	-102.8
		^{97}Mo (I = 5/2, 9.55%)	1	48.5	40.8	54.4
		^{183}W (I = 1/2, 14.31%)	3	-30.6	-25.7	-34.3
		^{33}S (I = 3/2, 0.76%)	6	13.1	13.0	14.9
$MoSe_2$	Mo_{Se}	^{95}Mo (I = 5/2, 15.92%)	1	-137.9	-65.1	-138.0
		^{97}Mo (I = 5/2, 9.55%)	1	-140.8	-66.5	-140.9
		^{95}Mo (I = 5/2, 15.92%)	3	19.6	11.1	20.7
		^{97}Mo (I = 5/2, 9.55%)	3	20.0	11.3	21.2
		^{77}Se (I = 1/2, 7.63%)	6	59.5	59.1	74.2
	W_{Se}	^{183}W (I = 1/2, 14.31%)	1	332.9	253.0	333.0
		^{95}Mo (I = 5/2, 15.92%)	3	14.6	8.4	16.5
		^{97}Mo (I = 5/2, 9.55%)	3	14.9	8.6	16.8
		^{77}Se (I = 1/2, 7.63%)	6	68.2	65.4	78.2
WSe2	W_{Se}	^{183}W (I = 1/2, 14.31%)	1	302.3	218.4	302.4
		^{183}W (I = 1/2, 14.31%)	3	-31.6	-25.5	-31.7
		^{77}Se (I = 1/2, 7.63%)	6	71.7	70.3	80.3
	Mo_{Se}	^{95}Mo (I = 5/2, 15.92%)	1	-120.4	-42.0	-120.4
		^{97}Mo (I = 5/2, 9.55%)	1	57.3	48.0	61.5
		^{183}W (I = 1/2, 14.31%)	3	-36.2	-30.3	-38.8
		^{77}Se (I = 1/2, 7.63%)	6	61.4	61.3	72.5
$MoTe_2$	Mo_{Te}	^{95}Mo (I=5/2, 15.92%)	1	-160.9	-150.8	-183.4
		^{97}Mo (I=5/2, 9.55%)	1	-164.3	-153.9	-187.2
		^{95}Mo (I=5/2, 15.92%)	3	0.1	0.1	-0.1
		^{97}Mo (I=5/2, 9.55%)	3	0.1	0.1	-0.1
		^{77}Te (I=1/2, 0.89%)	6	-7.8	-5.3	-8.7
		^{77}Te (I=1/2, 7.07%)	6	-9.4	-6.3	-10.5

References

1. Jain, A. *et al.* Commentary: The Materials Project: A materials genome approach to accelerating materials innovation. *APL Materials* **1**, 011002 (2013).
